# The effectiveness of ibandronate in reducing the risk of nonvertebral fractures in women with osteoporosis: systematic review and meta-analysis of observational studies

**DOI:** 10.1007/s11096-023-01666-x

**Published:** 2023-12-19

**Authors:** Carlos Alves, Diogo Mendes, Ana Penedones, Tânia Oliveira, António Donato, Francisco Batel-Marques

**Affiliations:** 1 Laboratory of Social Pharmacy and Public Health, Faculty of Pharmacy, Polo Ciencias da Saude, University of Coimbra, Azinhaga de Santa Comba, Celas, 3000-548 Coimbra, Portugal; 2 Clevidence, Lda., Taguspark, Oeiras, Portugal; 3 Tecnimede, SA, Sintra, Portugal

**Keywords:** Ibandronic acid, Meta-analysis, Observational study, Osteoporosis, Osteoporotic fractures, Systematic review

## Abstract

**Background:**

Ibandronate is effective in reducing the risk of vertebral fractures, but experimental evidence offers conflicting results regarding nonvertebral fractures. Real-world evidence has been published evaluating the anti-nonvertebral fracture effect of ibandronate.

**Aim:**

This meta-analysis of observational studies assessed the effectiveness of ibandronate in reducing the risk of nonvertebral fractures in women with osteoporosis.

**Method:**

Pubmed/Embase databases were searched for observational studies. Risks of nonvertebral fractures and hip fractures were the outcomes. Meta-analyses were performed pooling rate ratios (RRs), using random-effects models. Data were reanalysed in sensitivity analyses considering Knapp–Hartung method and Bayesian random-effects.

**Results:**

Six cohort studies were included. Overall, once-monthly 150 mg oral ibandronate reduced the risk of nonvertebral fractures (RR 0.84; 95% CI 0.76–0.94). Similar results were obtained when the comparison was restricted to once-monthly 150 mg risedronate, but no differences were found when the comparator was other oral bisphosphonates (weekly alendronate/risedronate). Ibandronate didn’t significantly change the risk of hip fractures (RR 1.25; 95% CI 0.89–1.76). The risk of hip fracture was comparable between once monthly, 150 mg oral ibandronate and other oral bisphosphonates. Intravenous ibandronate was not effective in reducing hip fractures comparing to intravenous zoledronate. The low number of studies diminished the robustness of sensitivity analyses.

**Conclusion:**

Results suggest that once-monthly 150 mg oral ibandronate may be as effective as other oral bisphosphonates in reducing the risk of nonvertebral fractures. However, uncertainty associated to the small number of included studies, which are characterized by heterogeneous demographics and methodologies, precluded definitive conclusions.

**Supplementary Information:**

The online version contains supplementary material available at 10.1007/s11096-023-01666-x.

## Impact statements

- Overall, once-monthly 150 mg oral ibandronate was associated with a reduction in the risk of overall nonvertebral fractures. Stratification of the results demonstrated that ibandronate may be as effective as other oral bisphosphonates.
- While the risk of hip fracture was comparable between once monthly 150 mg oral ibandronate and other oral bisphosphonates, intravenous (IV) ibandronate was not as effective as IV zoledronate.
- Although once-monthly 150 mg oral ibandronate seemed effective in preventing nonvertebral fractures, the small number of included studies, characterized by heterogeneous demographics and methodologies, diminished the robustness of sensitivity analyses, and precluded definitive conclusions.
- These findings highlight the need for further real-world studies to clarify the effectiveness of ibandronate compared to other bisphosphonates in the prevention of osteoporotic nonvertebral fractures.

## Introduction

Bisphosphonates are recommended as initial treatment for preventing osteoporotic fractures [1–4]. Ibandronate is indicated for the treatment of osteoporosis in women at increased risk of fracture, with the advantage of offering less frequent dosing intervals comparing to other oral bisphosphonates [5–7]. Two dosing regimens are approved: the once-monthly 150 mg tablet, and the 3 mg intravenous injection every 3 months [8].

Nonvertebral fractures are associated with high morbidity, health-related costs, and quality of life deterioration [9–11]. Current experimental evidence offers conflicting results on ibandronate preventing nonvertebral fractures. A post-hoc analysis of BONE trial concluded that ibandronate reduced the risk of nonvertebral fractures in a high-risk subgroup of patients (femoral neck BMD T score < − 3.0) [5]. One pooled analysis and one meta-analysis of randomized controlled trials (RCTs) demonstrated that high dose ibandronate (annual cumulative exposure [ACE] ≥ 10.8 mg) significantly reduced the risk of nonvertebral fractures [12, 13]. Though, meta-analyses of RCTs concluded that ibandronate was ineffective in preventing overall nonvertebral fractures, and hip and wrist fractures in particular [4, 14].

There are several observational studies evaluating the anti-nonvertebral fracture effect of ibandronate [15–17]. Observational data may contribute to clarify effectiveness of ibandronate in the prevention of nonvertebral fractures in populations under long-term treatment in clinical practice. However, it would be relevant to perform a meta-analysis of those studies, not only to assess the effectiveness of ibandronate in reducing the risk of nonvertebral fractures, but also to explore the existence of inconsistencies.

### Aim

The aim of this systematic review and meta-analysis of observational studies was to assess the effectiveness of ibandronate in reducing the risk of nonvertebral fractures in women with osteoporosis.

## Method

This systematic review and meta-analysis was conducted and reported according to the Centre for Reviews and Dissemination’s (CRD) guidance and the “Preferred Reported Items for Systematic Reviews and Meta-Analysis (PRISMA) 2020” [18, 19].

### Eligibility criteria

Studies were included if they met the following criteria:

- Population: women with osteoporosis;
- Intervention: ibandronate;
- Comparators: any treatment (other antiosteoporosis treatments, placebo, insufficient dosing regimen of ibandronate, or non-use of ibandronate);
- Outcomes: overall nonvertebral fractures; hip fractures;
- Study design: observational, controlled studies (cohort, case–control);
- Language: only English;
- Timing: no restrictions.

### Search strategy

Literature search followed the strategy from a systematic review evaluating ibandronate in preventing osteoporotic fractures [20]. The search strategy was applied to Pubmed and Embase, and duly updated through May 23rd, 2023 (Table S1). Bibliographic references lists of all relevant studies were hand searched to identify relevant studies.

### Study selection

Two researchers independently screened by hand the titles and abstracts and selected full articles for inclusion. Disagreement was resolved by discussion and consensus with a third researcher.

### Data collection

The following data were extracted from each study: reference, study design, population, intervention, comparator, outcomes, and results. Data were extracted from each included study by two researchers independently to a predeveloped form.

### Risk of bias assessment

The Risk of Bias In non-randomized Studies of Interventions (ROBINS-I) tool, developed by Cochrane Collaboration, was used to assess the risk of bias in non-randomized studies [21]. Each assessment study can be graded into one of five categories: low risk of bias, moderate risk of bias, serious risk of bias, critical risk of bias, and no information.

### Data analysis and data synthesis

A meta-analysis was performed by pooling rate ratios (RRs) with their 95% confidence intervals (CIs), using the DerSimonian and Laird random-effects model [22]. The most adjusted effect size estimate was used when more than one estimate was presented. Analyses were disaggregated according to different comparators. The I^2^ statistic test was used to assess for heterogeneity between studies, where an I^2^ > 50% was indicative of substantial heterogeneity [23]. Publication bias was assessed through funnel plots [24].

A sensitivity analysis was conducted to explore the robustness of the initial findings, using two methods to recalculate risks: a) the Knapp–Hartung method in combination with the Paule-Mandel estimator for the between-study variance [25]; b) a Bayesian random-effects meta-analysis [26]. A 95% prediction interval (PrI) was also estimated. The influence of the studies designs and methodological quality scores in the results was assessed.

Statistics were performed using R software (R version 4.1.2).

## Results

**Fig. 1:**
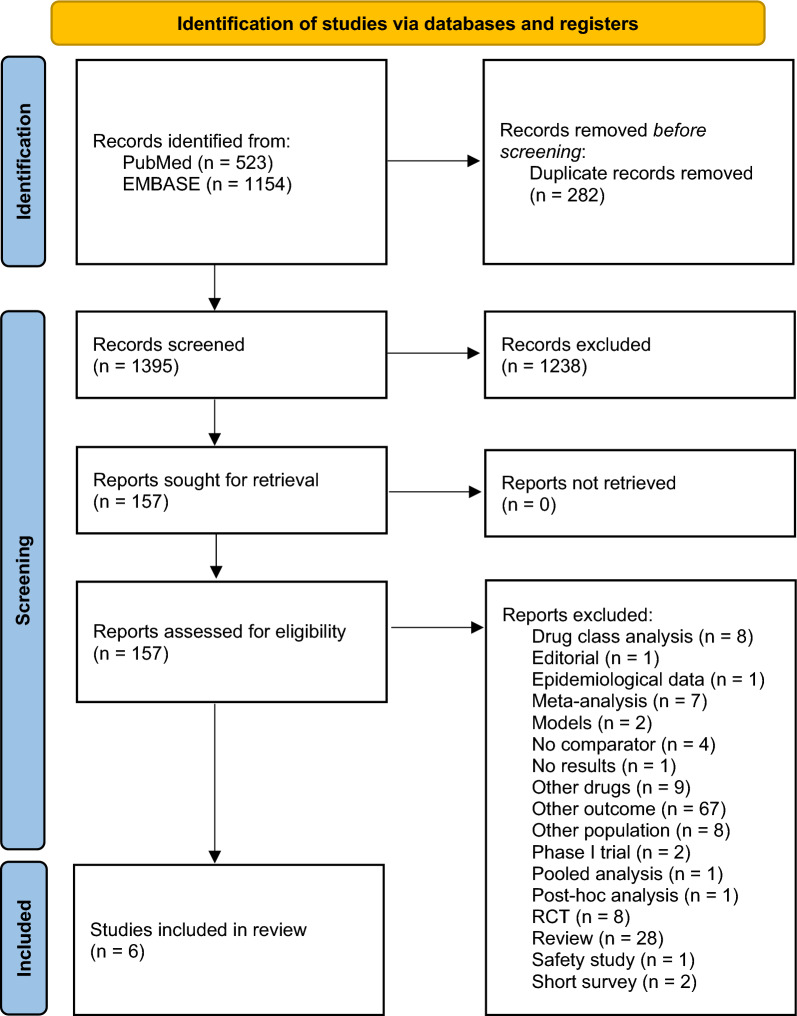
PRISMA flowchart

Figure 1 presents the flowchart of the systematic review of literature. A total of 1677 references were identified, of which 282 were considered duplicates. After reviewing titles and abstracts, further 1238 references were excluded. Lastly, 157 references were assessed for eligibility. From those, 6 observational studies were included [15–17, 27–29]. No studies were identified by hand search from reference lists.

### Characteristics of the studies

Table 1 describes the characteristics of the studies, which are all retrospective cohorts. One study was conducted in Korea, one study in Germany, and four in the United States of America (USA). The sample size ranged between 2166 and 95,802 patients, while the median time of follow-up varied from 0.6 to 3 years.

**Table 1:** Observational studies of ibandronate versus comparators for the prevention of fractures

Reference	Study design	Data sources (Country)	Time of follow-up	Population	Ibandronate	Control	Confounding adjustment, (number of covariates)	Previous fracture (%)	Outcomes
O’Kelly et al. [29]	Retrospective cohort	WIG2 benchmark database (Germany)	36 months [3 years] (time of analysis)	95,802 women aged ≥ 50 years who received an osteoporosis medication	IV ibandronate, infusion every 3 months (n = 1801)	No treatment (baseline: 3 months after the beginning of treatment)	Age (1)	IV ibandronate (14.4%)	Hip fracture
Lee and Lee [15]	Retrospective cohort	Korean National Health Insurance Service Senior Cohort (Korea)	384.1 ± 336.0 days [1.1 years] (mean time of follow-up [ibandronate group])	6908 women aged ≥ 65 years of age with primary osteoporosis initiating BP (without malignancy or Paget´s disease)	Oral ibandronate, 150 mg once monthly (n = 3454)	Oral BP (risedronate, 150 mg once monthly [n = 3454])	Propensity scores: age at the index date, household income level, history of fracture, Charlson comorbidity index, comorbidities, recent medications, health service use, and classification of the index date (6)	Ibandronate (45.9%), risedronate (45.8%)	Nonvertebral fracture, hip fracture
Yun et al. [16]	Retrospective cohort	Medicare (USA)	0.8–1.5 years (median times of follow-up, depending on the drug)	2166 women aged ≥ 65 years initiating various osteoporosis therapies	IV ibandronate, infusion every 3 months (n = 492)	IV zoledronate (zoledronate, infusion annually [n = 1674])	Propensity scores: age, gender, race, geography, income, osteoporosis related conditions, glucocorticoid-related disease, bone disease related, diabetics, renal disease, fall related conditions, cancer, acute myocardial infarction, depression, other heart problems and medications including hormone therapy at baseline (13)	IV ibandronate (13.6%), IV zoledronate (9.9%)	Hip fracture
Siris et al. [17]	Retrospective cohort	MarketScan Commercial Claims and Encounters and Medicare Supplemental and Coordinator of Benefits databases (USA)	872 days [2.4 years] (mean time of follow-up)	47,741 women aged ≥ 45 years under treatment with oral BP ≥ 12 months (without malignancy or Paget's disease)	Oral ibandronate, 150 mg once monthly (MPR 50%-79%; MPR ≥ 80%) †	No treatment (MPR < 50%)	Any fracture, rheumatoid arthritis diagnosis, smoking, number of physician office visits, number of prescriptions, use of oral glucocorticoids, and use of raloxifene or estrogen (7)	4.3%	Nonvertebral fracture
Abelson et al. [27]	Retrospective cohort	Ingenix Lab/Rx and Medstat MarketScan (USA)	12 months [1 year] (time of analysis)	14,288 women aged ≥ 65 years with primary osteoporosis (without malignancy or Paget´s disease)	Oral ibandronate, 150 mg once monthly (n = 14,288)	No treatment (baseline: 3 months after the beginning of treatment)	Age, history of prior fracture, glucocorticoid use, and diagnosis of rheumatoid arthritis (4)	Ibandronate (17%)	Nonvertebral fracture, hip fracture
Harris et al. [28]	Retrospective cohort	i3 Innovus and i3 IMPACT claims databases (USA)	7 months [0.6 years] (mean time of follow-up)	64,182 women aged ≥ 45 years with primary osteoporosis adherent to BP treatment (without malignancy or Paget´s disease)	Oral ibandronate, 150 mg once monthly (n = 7345)	Oral BP (alendronate, 35 mg or 70 mg per week, and risedronate 35 mg once weekly [n = 56837])	Age; osteoporosis diagnosis; use of dual energy X-ray absorptiometry; fracture history; number of concomitant medications; use of estrogen; and number of outpatient visits in the pre-index period (7)	Ibandronate (3.6%), bisphosphonates (3.7%)	Nonvertebral fracture, hip fracture

Legend: BP: bisphosphonates; IV: intravenous; MPR: medication possession ratio; USA: United States of America.

^†^ Sample with index ibandronate: n = 47,741; the number of patients in each MPR category is not provided in the publication

Four studies assessed the effectiveness of once-monthly 150 mg oral ibandronate and two studies assessed the effectiveness of the intravenous (IV) ibandronate (Table 1). The control group was the use of other bisphosphonates in three studies and “no treatment” in three studies.”No treatment” was defined as medication possession ratio [MPR] < 50% by Siris et al., and as the initial 3-month period of therapy by Abelson et al. and by O’Kelly et al. [17, 27, 29]. Two studies used propensity scores to adjust for confounders [15, 16]. The number of covariates considered for the adjustment varied between the studies, with Yun et al. considering 13 covariates for adjustment and O’Kelly et al. only considering one (age) [16, 29]. The risks of fractures were estimated using Cox proportional hazards models in four studies [15–17, 28], and simple proportions in two studies [27, 29].

Overall nonvertebral fractures were assessed in four studies and hip fractures in five (Table 1). Fracture outcomes were identified via International Classification of Diseases (ICD) codes in four studies [15, 17, 27, 28], and claims data and algorithms in two studies [16, 29].

### Risk of bias

**Fig. 2:**
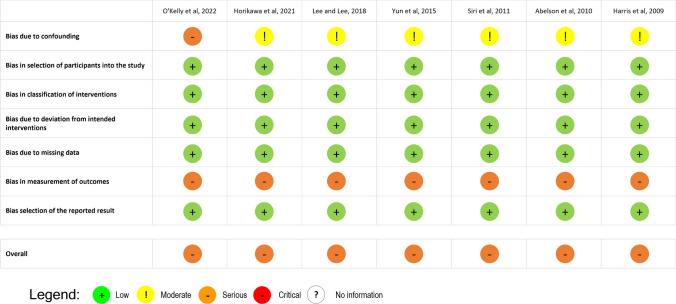
Risk of bias using the ROBINS-I tool

The results of the risk of bias assessment are illustrated in Fig. 2. All studies had serious risk of bias. Data on methods were not clear on how to avoid confounding in all studies. In addition, outcomes’ assessors were aware of the received intervention. However, it is not clear in which way this influenced the measurement of the outcome.

### Risk of nonvertebral and hip fractures

**Fig. 3:**
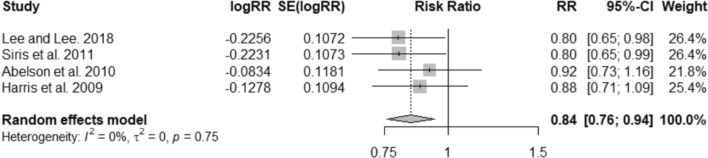
Meta-analysis of the risk of nonvertebral fractures

The findings suggest that, overall, once-monthly 150 mg oral ibandronate reduces the risk of nonvertebral fractures (RR 0.84; 95% CI 0.76–0.94; I^2^ = 0%) (Fig. 3). The results were similar when the comparison was restricted to once-monthly 150 mg risedronate (RR 0.80; 95% CI 0.65–0.98; I^2^ = NA) but did not remain statistically significant when the comparator chosen was “no treatment” (RR 0.85; 95% CI 0.73–1.00; I^2^ = 0%) or other oral bisphosphonates (alendronate 35 mg/70 mg weekly, or risedronate 35 mg weekly) (RR 0.88; 95% CI 0.71–1.09; I^2^ = NA) (Fig. S3).

**Fig. 4:**
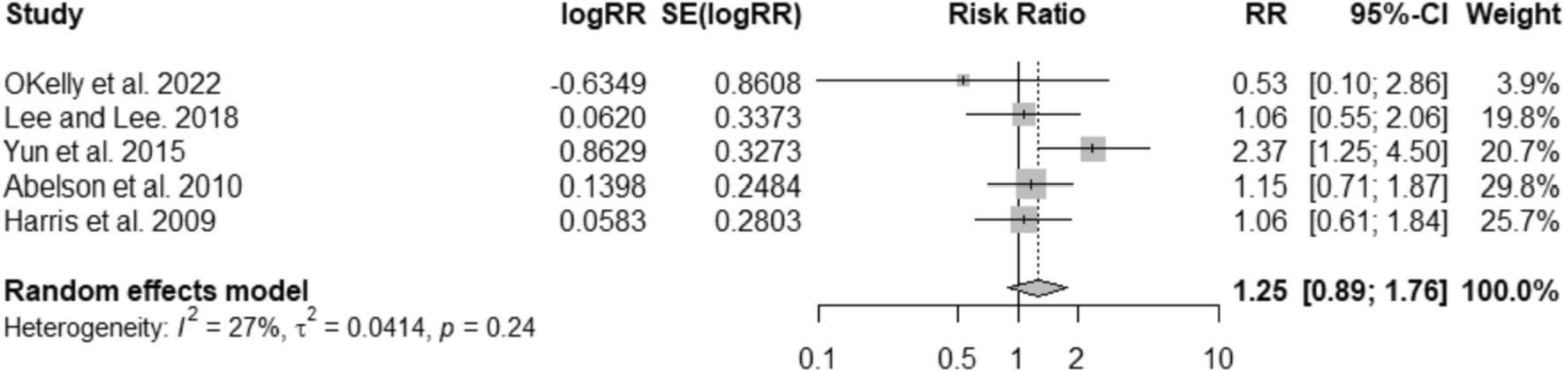
Meta-analysis of the risk of hip fractures

Ibandronate did not significantly change the risk of hip fractures (RR 1.25; 95% CI 0.89–1.76; I^2^ = 27%) (Fig. 4). The results remain similar when the comparison was restricted to other oral bisphosphonates (alendronate 35 mg/70 mg weekly, or risedronate 35 mg weekly) (RR 1.06; 95% CI 0.61–1.84; I^2^ = NA), 150 mg oral risedronate (RR 1.06; 95% CI 0.55–2.06; I^2^ = NA), or “no treatment” (RR 1.08; 95% CI 0.68–1.73; I^2^ = 0%). Comparing to IV zoledronic acid, IV ibandronate is not effective in reducing hip fractures (RR 2.37; 95% CI 1.15–4.50; I^2^ = NA) (Fig. S4).

### Publication bias

No significant asymmetry was identified in the funnel plots of both meta-analyses (Figs. S1 and S2). The low number of studies limited these analyses.

### Sensitivity analysis

According to the Knapp–Hartung method, the risk of nonvertebral fractures did not change; the 95% PrI was estimated as RR: 0.67–1.07 (Fig. S5). The risk of hip fracture did not significantly change comparing to the initial analysis (RR 1.25; 95% CI 0.76–2.06); the 95% PrI is wide (RR 95% PrI 0.51–3.09) (Fig. S6).

The results reported by the Bayesian meta-analyses did not identify statistically significant protective effects from ibandronate [nonvertebral fractures RR 0.85; 95% credible interval (CrI) 0.56–1.29); hip fractures RR 1.20; 95% CrI 0.66–2.20] (Figs. S7 and S8).

No further sensitivity analyses were conducted since all studies have similar design and methodological quality scores.

## Discussion

This meta-analysis of observational studies evaluates the effectiveness of ibandronate in preventing osteoporotic nonvertebral fracture. There are reasons why this study stands out from a previous systematic review evaluating the antifracture benefit of ibandronate [20]. First, this meta-analysis is supported by an updated literature search which identified a new study [29]. Updating systematic reviews is a recommended procedure in clinical investigation, as newly identified studies can change the conclusions of previous publications [30]. Disregarding new evidence can threaten the validity of systematic reviews and misleading healthcare professionals, patients, and regulatory authorities at the time of decision-making [31, 32]. Second, the previous systematic review performed a qualitative evaluation of ibandronate antifracture effect. This meta-analysis provides a weighted average of the results of all individual studies and conducts a sensitivity analysis to assess the robustness of the results, helping readers understanding the conclusions. Statistical synthesis of the evidence adds value to this research topic since it provides objective risk estimates rather than qualitative descriptions [33].

RCTs provide the most robust evidence regarding the efficacy and short-term safety of pharmacological interventions. Among their advantages, the strict inclusion/exclusion criteria, random allocation, continuous monitoring of patients, and precise definition of endpoints reduces the risk of bias and confounding [34]. However, real-world studies are essential to provide evidence of treatment effectiveness, by including patient populations that may be representative of clinical practice, larger sample sizes, and extended follow-up times when compared to RCTs. In this context, evidence of efficacy and safety of bisphosphonates from RCTs may not predict their actual effectiveness in clinical practice because of clinical, demographic, and suboptimal persistence/adherence differences between the populations. Therefore, there is a rational to conduct a meta-analysis of observational studies evaluating the effectiveness of ibandronate in preventing osteoporotic nonvertebral fractures. Moreover, meta-analyses of nonexperimental studies are frequently designed to explore eventual sources of heterogeneity among studies rather than to find evidence of causative associations [35].

These results suggest that ibandronate reduces the risk of nonvertebral fractures in general. Regarding this outcome, all the included studies evaluated ibandronate in reducing nonvertebral fractures as a once-monthly 150 mg oral regimen. However, the analysis of the results should take into consideration the different comparators used in the studies. The sensitivity analysis demonstrated that once-monthly 150 mg oral ibandronate reduced the risk of nonvertebral fractures compared with once-monthly 150 mg oral risedronate [15]. No differences were found when once-monthly 150 mg oral ibandronate was compared to weekly risedronate or weekly alendronate [28]. The comparison between ibandronate and “no treatment” returned a reduced risk, but without statistical significance [17, 27].

Although the results did not demonstrate an overall risk reduction of hip fracture, oral ibandronate seems to have a comparable risk of hip fracture to other oral bisphosphonates. No risk differences were found between once-monthly 150 mg oral ibandronate versus the other oral bisphosphonates (either once-monthly 150 mg oral risedronate, or weekly risedronate or weekly alendronate), nor versus “no treatment” [15, 27, 28]. However, the results suggest that IV zoledronate (once yearly) is more effective than IV ibandronate (once quarterly) to prevent hip fractures [16].

Both risedronate and alendronate are approved to prevent hip fractures in women with osteoporosis [36, 37]. Since the risk of hip fractures was comparable between once-monthly 150 mg oral ibandronate and the other oral bisphosphonates (once-monthly 150 mg oral risedronate, or weekly risedronate, and weekly alendronate), whether ibandronate could also be recommended to prevent such type of fracture may be matter of discussion. These results are in line with those from previous meta-analyses of RCTs, where no differences were found between these three bisphosphonates on the risk of hip fracture [38, 39]. Therefore, this meta-analysis of observational studies may contribute to clarifying the comparative effectiveness of ibandronate versus other bisphosphonates in the prevention of nonvertebral fractures in real-world clinical practice.

This study assessed hip fractures as the only outcome for site-specific fractures. Hip fractures are one of most severe type of osteoporotic fractures and are associated with higher mortality, reduced quality of life, and increased health resources consumption [40]. As hip fractures are an important endpoint considered in the design of RCTs of new antiosteoporosis drugs, studies assessing the effectiveness of the bisphosphonates in reducing this type of fractures in real-world clinical practice are valued by decision-makers [32, 41, 42]. Furthermore, only the study of O’Kelly et al. reported results for other type of fracture (wrist/forearm fracture) [29]. Given this, it was not possible to conduct a meta-analysis including only one study.

A strength of this meta-analysis is including only studies reporting results on definite nonvertebral fracture outcomes, and not studies reporting results for surrogate-type endpoints (e.g., changes in BMD). Although the incidence of osteoporotic fractures is usually associated with changes in BMD, this is still a predictive risk factor that do not replace the measurement of definite fracture outcomes [34, 35]. The marketing authorisations of bisphosphonates to prevent fractures in osteoporosis were granted based on statistically significant risk reductions of definite fracture outcomes (rather than solely on changes in BMD) [36]. Therefore, the summary of product characteristics of ibandronate highlights that the efficacy of the drug is yet to be proved in the prevention of nonvertebral fractures.

Some limitations must be considered. First, only 6 studies verified inclusion criteria. There are several observational studies evaluating the effectiveness of bisphosphonates published in the scientific literature, but without disaggregating the results at drug level [37]. A thorough literature search has been conducted but only a small of group studies evaluating the risk of nonvertebral fractures associated with ibandronate were found. Second, the studies used different control groups. Not only different bisphosphonates were used as active comparators but also three studies comparing ibandronate with “no treatment” adopted different approaches when defining the control groups. In the study of Siris et al. patients with a medication possession ratio (MPR) of less than 50% were considered the referent ‘‘untreated’’ population [17]. The studies of O’Kelly et al. and Abelson and et al. evaluated the risk of nonvertebral fractures by comparing the incidence of those fractures during an initial 3-month period of therapy with the incidence of fractures observed in the subsequent first year of therapy [27, 29]. Authors argued that the baseline fracture incidence during the initial 3 months of therapy may accurately reflect the underlying risk of the cohort, as bisphosphonates may take up to 3 months to reach maximum effectiveness [38]. The different design approaches between the studies using “no treatment” as a control group may be the reason why they did not reach concordant nonvertebral fracture risk estimates, resulting in a non-statistically significant risk reduction in the meta-analysis. Third, the results were only stratified according to different comparators, with all studies having the same design and risk of bias assessment. The influence of additional risk factors in the results needs to be accounted for when analysing these findings. However, most studies did not detail the results according to known risk factors for nonvertebral fractures, like age, prior use of bisphosphonates, and previous fractures. Fourth, there is no currently satisfactory methodology to perform meta-analysis including a small number of studies, particularly because between-studies heterogeneity cannot be reliably estimated. The initial analysis heterogeneity was most likely underestimated by the frequentist method. Therefore, additional analyses were conducted to better account the uncertainty. The Knapp–Hartung method (combined with the Paule-Mandel estimator) is recommended by Cochrane Collaboration for this specific scenario and the Bayesian random-effects meta-analysis is an alternative approach when having a low number of studies [25]. Fifth, the covariates used for the adjustment and approaches to control confounders varied significantly between the studies. Therefore, studies were assessed as having low methodological quality. Sixth, there are additional differences between the studies regarding their methods and demographics. Three studies included patients aged ≥ 45 years old, while three considered patients aged ≥ 65 years old. Additionally, sample sizes, median time of follow-up, and proportion of patients with previous fracture varied greatly between studies. While the heterogeneity found in these meta-analyses was not excessive (maximum I^2^ = 27%), the possibility of studies’ demographic and methodological discrepancies have influence in the risk estimates should not be ruled out. Seventh, we only searched Pubmed and Embase. However, these are the two most comprehensive biomedical literature databases and comprised more than 35 and 44 million citations, respectively [49, 50]. Eight, no protocol of this study was previously published, which could increase transparency and reduce potential for bias. Nonetheless, guidance to conduct and report this meta-analysis was followed.

## Conclusion

These results suggest that once-monthly 150 mg oral ibandronate may be as effective as other oral bisphosphonates in reducing the risk of nonvertebral fractures. However, the small number of included studies are associated with uncertainty since they have heterogeneous demographics and methodologies. Thus, it was not possible to conduct a thorough assessment of the consistency of these findings and precaution is needed before taking definitive conclusions and offering recommendations. Further real-world studies are needed to clarify the effectiveness of ibandronate compared to other bisphosphonates in the prevention of osteoporotic nonvertebral fractures.

### Supplementary Information

Below is the link to the electronic supplementary material.

Supplementary file1 (DOCX 2038 KB)
