# Supplementary material for: The effectiveness of ibandronate in reducing the risk of nonvertebral fractures in women with osteoporosis: systematic review and meta-analysis of observational studies
Source: Int J Clin Pharm. 2023 Dec 19;46(2):357–67. doi: 10.1007/s11096-023-01666-x (PMC10960777; doi:10.1007/s11096-023-01666-x)
Supplement: Supplementary file 1 — Supplementary file1 (DOCX 2038 KB) [file 11096_2023_1666_MOESM1_ESM.docx]

**Supplemental Figure 1**: Funnel plot for the studies included in the meta-analysis of the risk of nonvertebral fractures

**Supplemental Figure 2**: Funnel plot for the studies included in the meta-analysis of the risk of hip fractures


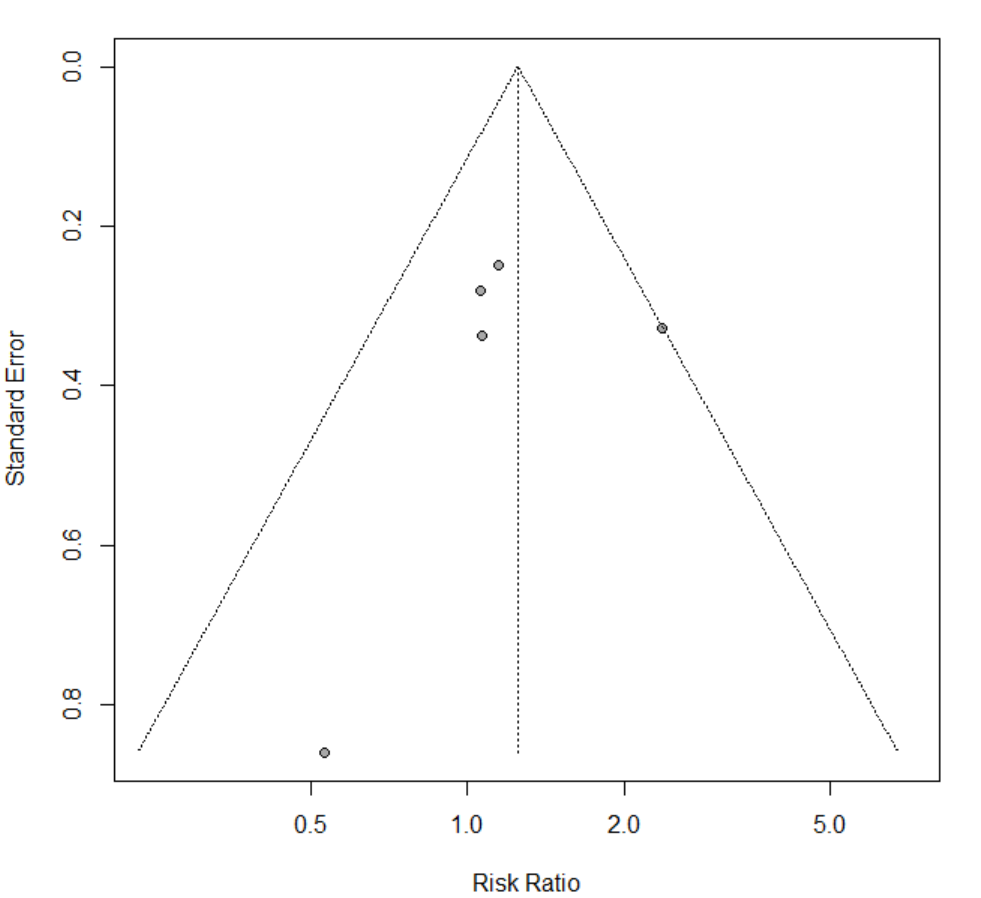


**Supplemental Figure 3**: Meta-analysis of the risk of nonvertebral fractures, stratified by different comparators.


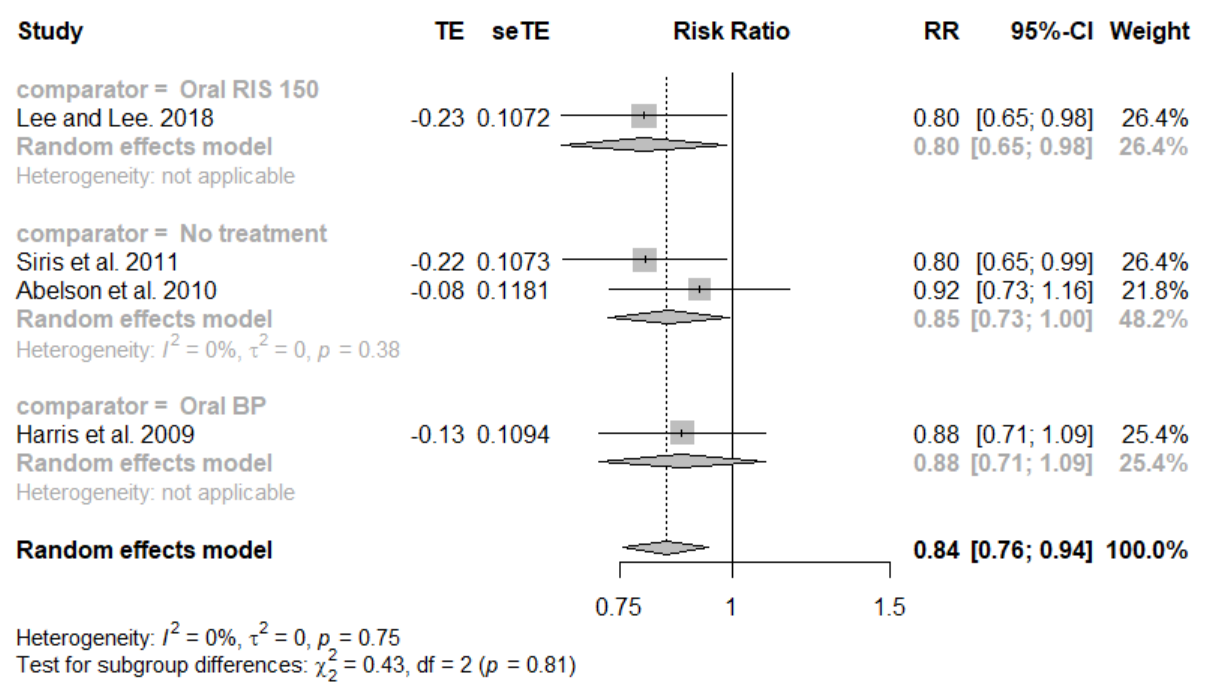


Legend: RIS, risedronate; BP, bisphosphonates

**Supplemental Figure 4:** Meta-analysis of the risk of hip fractures, stratified by different comparators.


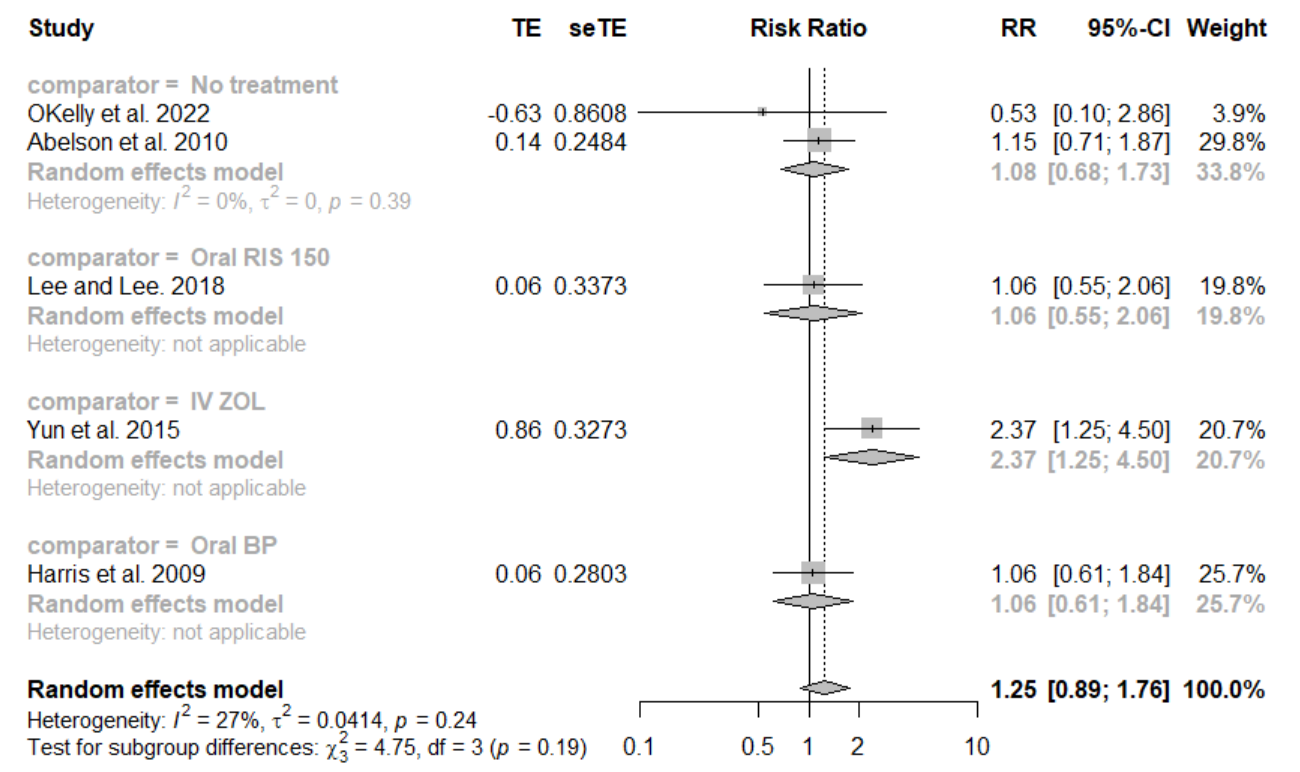


Legend: RIS, risedronate; BP, bisphosphonates; ZOL, zoledronic acid; IV, intravenous

**Supplemental Figure 5**: Meta-analysis of the risk of nonvertebral fractures according to the Knapp-Hartung method


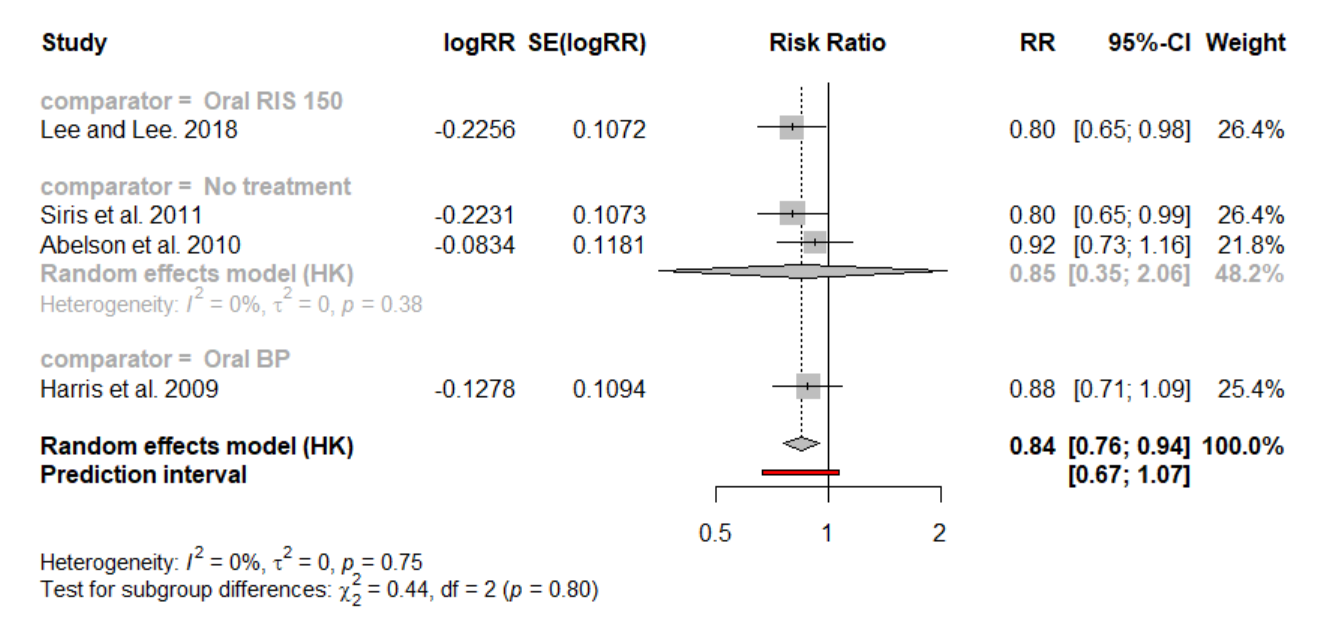


Legend: RIS, risedronate; BP, bisphosphonates;

**Supplemental Figure 6**: Meta-analysis of the risk of hip fractures according to the Knapp-Hartung method

**
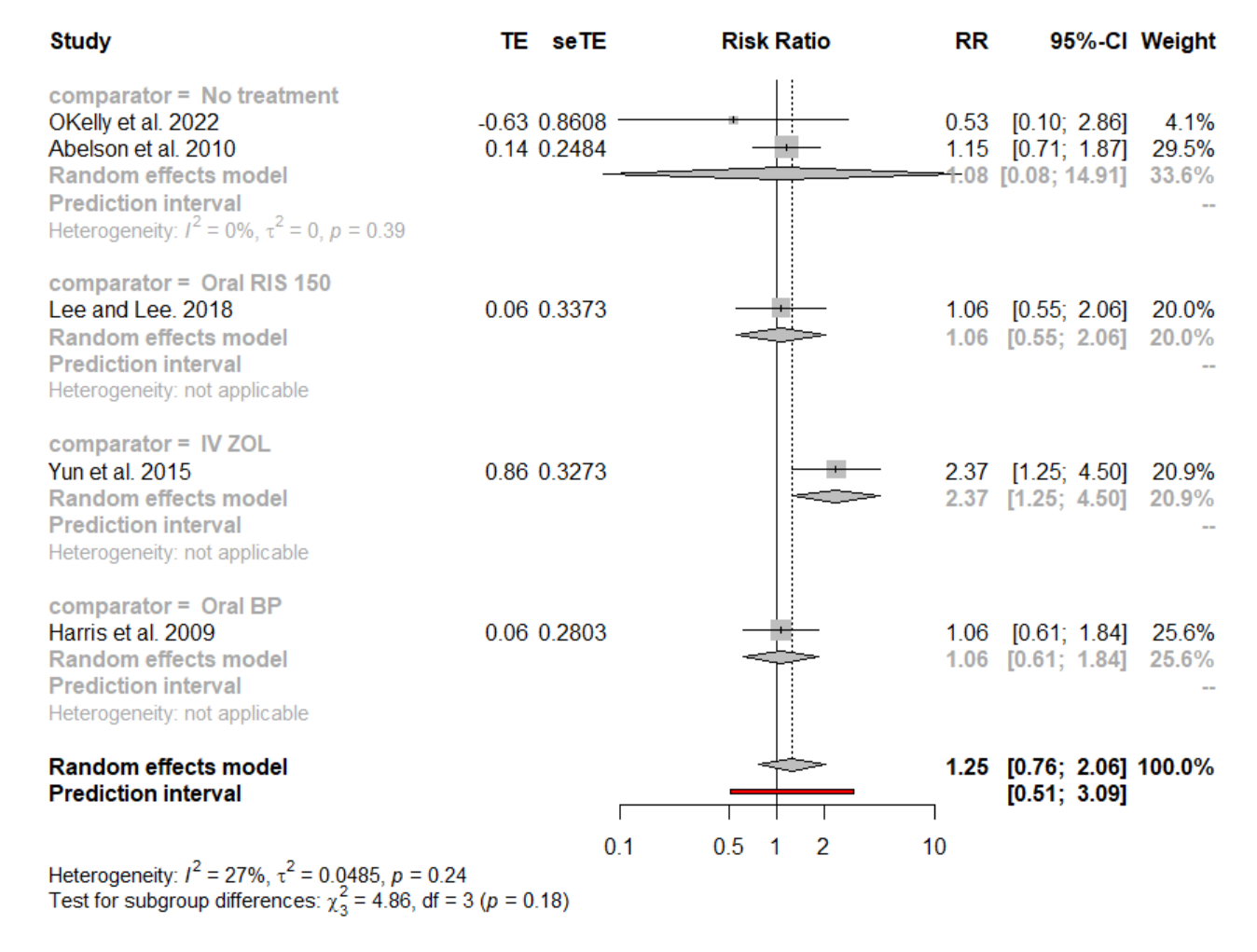
**

Legend: RIS, risedronate; BP, bisphosphonates; ZOL, zoledronic acid; IV, intravenous

**Supplemental Figure 7**: Bayesian meta-analysis of the risk of nonvertebral fractures

**
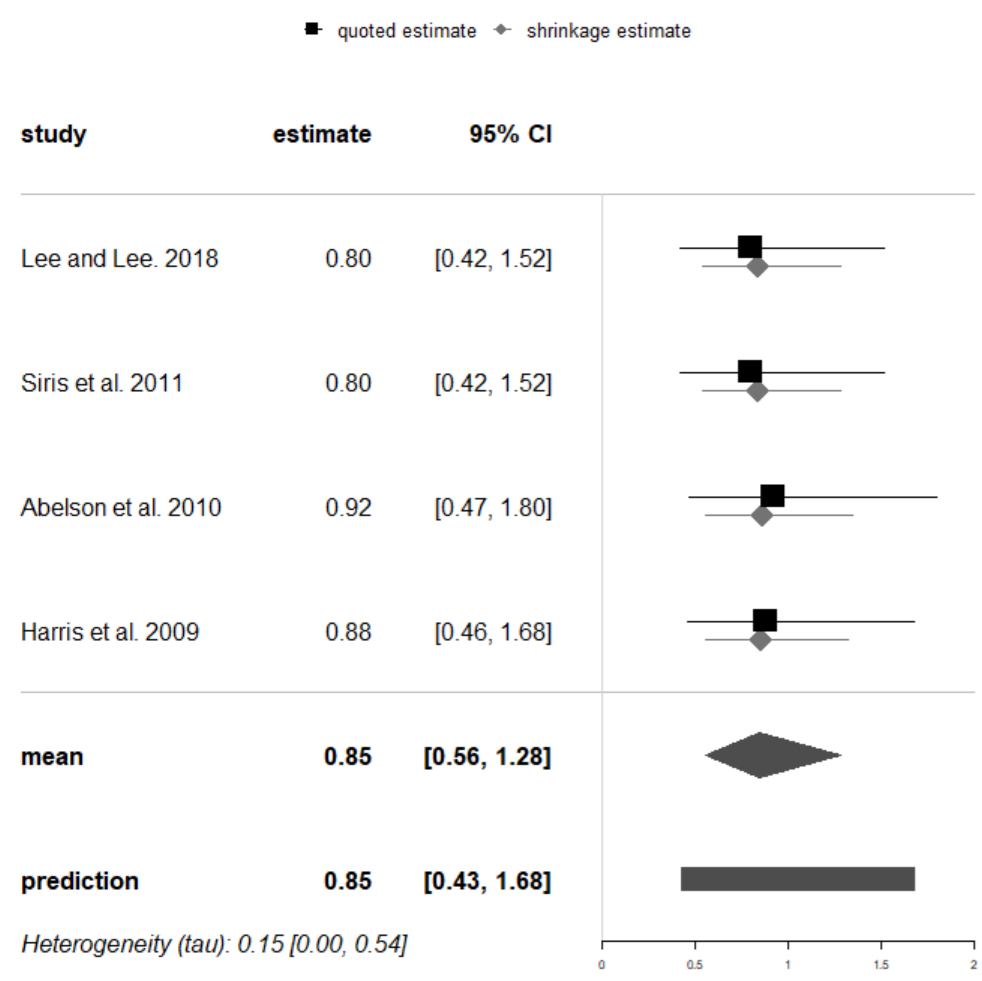
**

**Supplemental Figure 8**: Bayesian meta-analysis of the risk of hip fractures

**
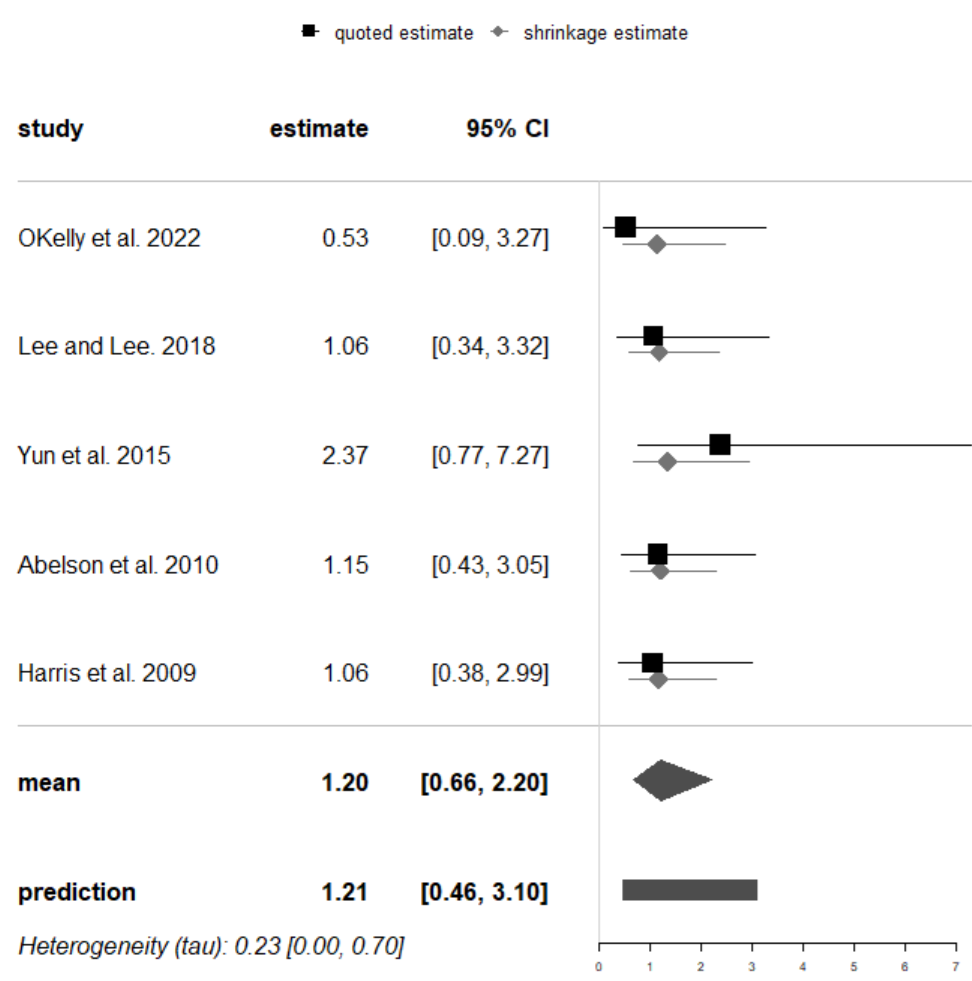
**

**Supplemental Table 1:** Literature Search Strategies

Bibliographic database: PubMed (<https://pubmed.ncbi.nlm.nih.gov/>)

Date: Since database’s inception until May 23, 2023.

| **Search** | **Equation** |
| --- | --- |
| 1 | "ibandronic acid"[MeSH Terms] OR ("ibandronic"[All Fields] AND "acid"[All Fields]) OR "ibandronic acid"[All Fields] |
| 2 | "ibandronic acid"[MeSH Terms] OR ("ibandronic"[All Fields] AND "acid"[All Fields]) OR "ibandronic acid"[All Fields] OR "ibandronate"[All Fields] OR "ibandronic"[All Fields] |
| 3 | 1 OR 2 |
| 4 | "randomized controlled trial"[Publication Type] OR "randomized controlled trials as topic"[MeSH Terms] OR "randomized controlled trial"[All Fields] OR "randomised controlled trial"[All Fields] |
| 5 | "random allocation"[MeSH Terms] OR ("random"[All Fields] AND "allocation"[All Fields]) OR "random allocation"[All Fields] |
| 6 | "double blind method"[MeSH Terms] OR ("double blind"[All Fields] AND "method"[All Fields]) OR "double blind method"[All Fields] OR ("double"[All Fields] AND "blind"[All Fields] AND "method"[All Fields]) OR "double blind method"[All Fields] |
| 7 | "single blind method"[MeSH Terms] OR ("single blind"[All Fields] AND "method"[All Fields]) OR "single blind method"[All Fields] OR ("single"[All Fields] AND "blind"[All Fields] AND "method"[All Fields]) OR "single blind method"[All Fields] |
| 8 | "clinical trial"[Publication Type] OR "clinical trials as topic"[MeSH Terms] OR "clinical trial"[All Fields] |
| 9 | "clinical trial, phase i"[Publication Type] OR "clinical trials, phase i as topic"[MeSH Terms] OR "clinical trial phase i"[All Fields] OR "phase i clinical trial"[All Fields] |
| 10 | "clinical trial, phase ii"[Publication Type] OR "clinical trials, phase ii as topic"[MeSH Terms] OR "clinical trial phase ii"[All Fields] OR "phase ii clinical trial"[All Fields] |
| 11 | "clinical trial, phase iii"[Publication Type] OR "clinical trials, phase iii as topic"[MeSH Terms] OR "clinical trial phase iii"[All Fields] OR "phase iii clinical trial"[All Fields] |
| 12 | "clinical trial, phase iv"[Publication Type] OR "clinical trials, phase iv as topic"[MeSH Terms] OR "clinical trial phase iv"[All Fields] OR "phase iv clinical trial"[All Fields] |
| 13 | "controlled clinical trial"[Publication Type] OR "controlled clinical trials as topic"[MeSH Terms] OR "controlled clinical trial"[All Fields] |
| 14 | "multicenter study"[Publication Type] OR "multicenter studies as topic"[MeSH Terms] OR "multicenter study"[All Fields] OR "multicentre study"[All Fields] |
| 15 | OR/4-14 |
| 16 | "clinical study"[Publication Type] OR "clinical studies as topic"[MeSH Terms] OR "clinical study"[All Fields] |
| 17 | "epidemiologic studies"[MeSH Terms] OR ("epidemiologic"[All Fields] AND "studies"[All Fields]) OR "epidemiologic studies"[All Fields] |
| 18 | "case control studies"[MeSH Terms] OR ("case control"[All Fields] AND "studies"[All Fields]) OR "case control studies"[All Fields] OR ("case"[All Fields] AND "control"[All Fields] AND "studies"[All Fields]) OR "case control studies"[All Fields] |
| 19 | "cohort studies"[MeSH Terms] OR ("cohort"[All Fields] AND "studies"[All Fields]) OR "cohort studies"[All Fields] |
| 20 | "follow up studies"[MeSH Terms] OR ("follow up"[All Fields] AND "studies"[All Fields]) OR "follow up studies"[All Fields] OR ("follow"[All Fields] AND "up"[All Fields] AND "studies"[All Fields]) OR "follow up studies"[All Fields] |
| 21 | "observational study"[Publication Type] OR "observational studies as topic"[MeSH Terms] OR "observational studies"[All Fields] |
| 22 | "longitudinal studies"[MeSH Terms] OR ("longitudinal"[All Fields] AND "studies"[All Fields]) OR "longitudinal studies"[All Fields] |
| 23 | "retrospective studies"[MeSH Terms] OR ("retrospective"[All Fields] AND "studies"[All Fields]) OR "retrospective studies"[All Fields] |
| 24 | "prospective studies"[MeSH Terms] OR ("prospective"[All Fields] AND "studies"[All Fields]) OR "prospective studies"[All Fields] |
| 25 | "cross sectional studies"[MeSH Terms] OR ("cross sectional"[All Fields] AND "studies"[All Fields]) OR "cross sectional studies"[All Fields] OR ("cross"[All Fields] AND "sectional"[All Fields] AND "studies"[All Fields]) OR "cross sectional studies"[All Fields] |
| 26 | OR/16-25 |
| 27 | 15 OR 26 |
| 28 | "case reports"[Publication Type] OR "case report"[All Fields] |
| 29 | "letter"[Publication Type] OR "correspondence as topic"[MeSH Terms] OR "letter"[All Fields] |
| 30 | "historical article"[Publication Type] OR "historical article"[All Fields] |
| 31 | "congress"[Publication Type] OR "congresses as topic"[MeSH Terms] OR "conference"[All Fields] |
| 32 | OR/28-31 |
| 33 | 27 OR 32 |
| 34 | 3 AND 33 |

Bibliographic database: EMBASE (<https://www.embase.com>)

Date: Since database’s inception until May 23, 2023.

| **Search** | **Equation** |
| --- | --- |
| 1 | 'ibandronic acid'/exp OR 'ibandronic acid' |
| 2 | 'ibandronate'/exp OR ibandronate |
| 3 | 1 OR 2 |
| 4 | 'randomized controlled trial (topic)'/exp OR 'randomized controlled trial (topic)' |
| 5 | 'randomization'/exp OR randomization |
| 6 | 'double blind procedure'/exp OR 'double blind procedure' |
| 7 | 'single blind procedure'/exp OR 'single blind procedure' |
| 8 | 'clinical trial'/exp OR 'clinical trial' |
| 9 | 'controlled clinical trial'/exp OR 'controlled clinical trial' |
| 10 | 'multicenter study'/exp OR 'multicenter study' |
| 11 | OR/4-10 |
| 12 | 'clinical study'/exp OR 'clinical study' |
| 13 | 'case control study'/exp OR 'case control study' |
| 14 | 'cohort analysis'/exp OR 'cohort analysis' |
| 15 | 'observational study'/exp OR 'observational study' |
| 16 | 'longitudinal study'/exp OR 'longitudinal study' |
| 17 | 'retrospective study'/exp OR 'retrospective study' |
| 18 | 'prospective study'/exp OR 'prospective study' |
| 19 | 'cross-sectional study'/exp OR 'cross-sectional study' |
| 20 | OR/12-19 |
| 21 | 11 OR 20 |
| 22 | 'case report'/exp OR 'case report' |
| 23 | 'abstract report'/exp OR 'abstract report' |
| 24 | 'letter'/exp OR letter |
| 25 | 'conference paper'/exp OR 'conference paper' |
| 26 | 'conference abstract'/exp OR 'conference abstract' |
| 27 | 'conference review'/exp OR 'conference review' |
| 28 | 'note'/exp OR note |
| 29 | 'editorial'/exp OR editorial |
| 30 | 'questionnaire'/exp OR questionnaire |
| 31 | OR/22-30 |
| 32 | 21 NOT 31 |
| 33 | 3 AND 32 |
| 34 | 'review'/exp OR review |
| 35 | systematic AND ('review'/exp OR review) |
| 36 | 'meta analysis'/exp OR 'meta analysis' |
| 37 | #34 OR #35 OR #36 |
| 38 | #32 NOT #37 |
| 39 | #3 AND #38 |
